# Bioengineered materials with selective antimicrobial toxicity in biomedicine

**DOI:** 10.1186/s40779-023-00443-1

**Published:** 2023-02-24

**Authors:** Pooyan Makvandi, Hao Song, Cynthia K. Y. Yiu, Rossella Sartorius, Ehsan Nazarzadeh Zare, Navid Rabiee, Wei-Xi Wu, Ana Cláudia Paiva-Santos, Xiang-Dong Wang, Cheng-Zhong Yu, Franklin R. Tay

**Affiliations:** 1 The Quzhou Affiliated Hospital of Wenzhou Medical University, Quzhou People’s Hospital, Quzhou, 324000 Zhejiang China; 2 Istituto Italiano di Tecnologia, Centre for Materials Interfaces, Pontedera, 56025 Italy; 3 Australian Institute for Bioengineering and Nanotechnology, The University of Queensland, Brisbane, QLD 4072 Australia; 4 Paediatric Dentistry and Orthodontics, Faculty of Dentistry, The University of Hong Kong, Prince Philip Dental Hospital, Hong Kong SAR, China; 5 Institute of Biochemistry and Cell Biology (IBBC), National Research Council (CNR), 80131 Naples, Italy; 6 School of Chemistry, Damghan University, Damghan, 36716-45667 Iran; 7 School of Engineering, Macquarie University, Sydney, NSW 2109 Australia; 8 Centre for Molecular Medicine and Innovative Therapeutics, Murdoch University, Perth, WA 6150 Australia; 9 Department of Pharmaceutical Technology, Faculty of Pharmacy of the University of Coimbra, University of Coimbra, 3000-548 Coimbra, Portugal; 10 REQUIMTE/LAQV, Group of Pharmaceutical Technology, Faculty of Pharmacy of the University of Coimbra, University of Coimbra, 3000-548 Coimbra, Portugal; 11 Department of Pulmonary and Critical Care Medicine, Zhongshan Hospital, Fudan University Shanghai Medical College, Shanghai, 200032 China; 12 School of Chemistry and Molecular Engineering, East China Normal University, Shanghai, 200241 China; 13 The Graduate School, Augusta University, Augusta, GA 30912 USA

**Keywords:** Antimicrobial nanotechnology, Immumodualtion, Selective toxicity, Smart nanomaterials, Targeting microorganism

## Abstract

Fungi and bacteria afflict humans with innumerous pathogen-related infections and ailments. Most of the commonly employed microbicidal agents target commensal and pathogenic microorganisms without discrimination. To distinguish and fight the pathogenic species out of the microflora, novel antimicrobials have been developed that selectively target specific bacteria and fungi. The cell wall features and antimicrobial mechanisms that these microorganisms involved in are highlighted in the present review. This is followed by reviewing the design of antimicrobials that selectively combat a specific community of microbes including Gram-positive and Gram-negative bacterial strains as well as fungi. Finally, recent advances in the antimicrobial immunomodulation strategy that enables treating microorganism infections with high specificity are reviewed. These basic tenets will enable the avid reader to design novel approaches and compounds for antibacterial and antifungal applications.

## Background

Although antibiotics have been developed for combating infectious diseases, microbial resistance remains a consistent global challenge [1–3]. The resistance developed by microorganisms through their defense systems threatens human health by generating resistant strains that evade eradication by even the most advanced antibiotics [4]. Currently available antibiotics for combating microbial infections are rapidly becoming ineffective because of the development of drug-resistant microbial strains. Antimicrobial nanomaterials represent a rational approach to combating antibiotic-resistant microbes [5, 6]. An ideal strategy for tackling these challenging diseases is the development of smart antimicrobial materials with selective toxicity against specific infectious microorganisms [7]. In light of this, several researches were devoted to foe bioengineering of nanomaterials to tune and modify their antibacterial activity against a specific pathogen. Accordingly, a wide range of nanoparticles were functionalized with bioactive compounds to bring selective toxicity against bacteria and fungi. Selective toxicity is the ability of antimicrobials to kill or inhibit deleterious microbes only while preserving the vitality of host cells or the healthy microbiome [8].

**Fig. 1:**
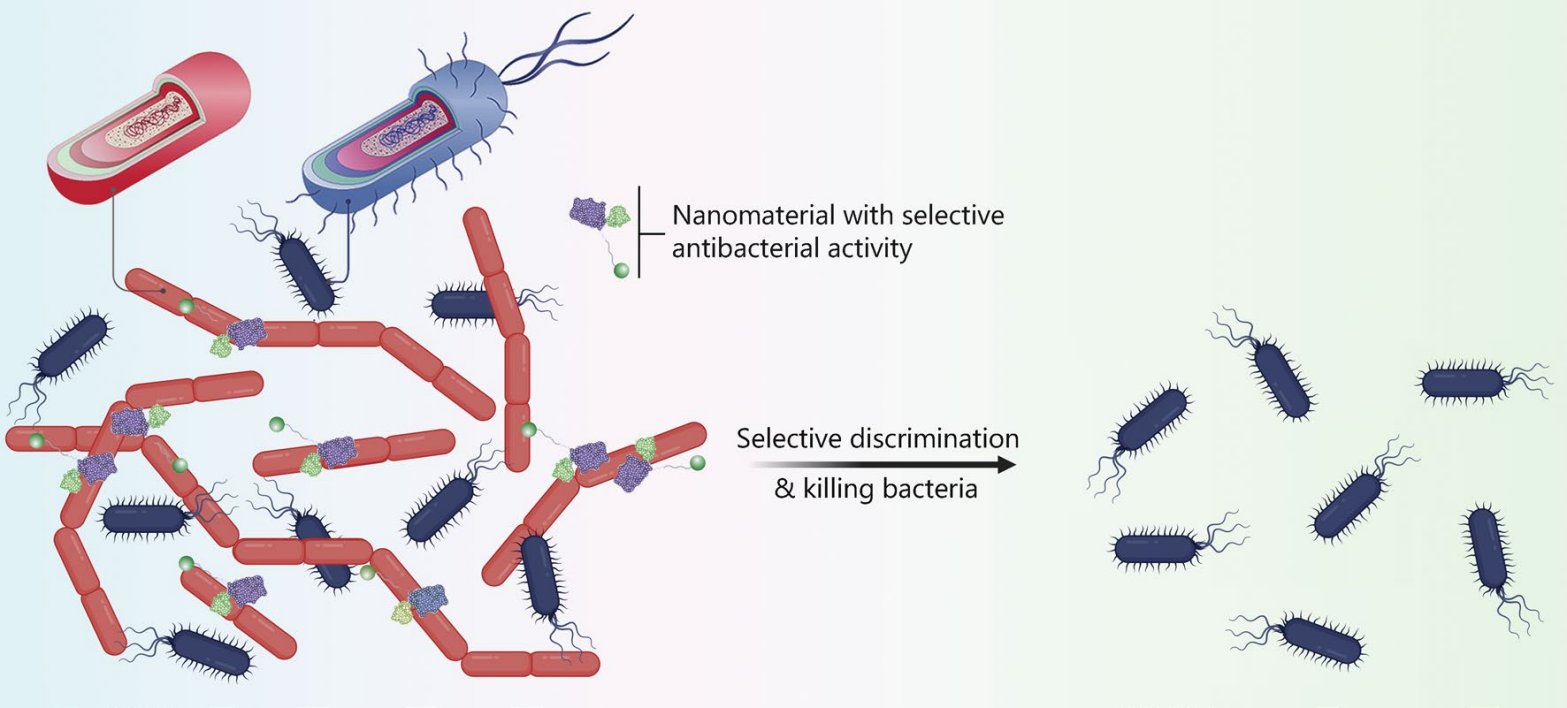
Schematic illustration of the selective toxicity of a material to combat specific microbes. The cell wall binding domains can selectively attach and kill pathogenic bacteria. Due to the presence of the attached ligand to silver nanoparticles, the nanoplatform can specifically bind to the pathogen as targeting ligands to discriminate bacterial strains and impart selective antibacterial performance

The present review summarizes the design of smart materials that selectively target specific types of microorganisms (Fig. 1). The cell characteristics of microbes, including Gram-positive bacteria, Gram-negative bacteria and fungi, are presented along with a discussion of the antimicrobial mechanisms involved in combating these microorganisms. Readers are then introduced to the design of advanced materials with selectivity. Apart from direct microbial killing using smart nanomaterials, the most recent studies in treating microorganism infections through immune modulations are also summarized. Acquisition of such knowledge will offer important cues to the passionate reader on effective control of microbial infections with high selectivity and low side effects.

## Cell characteristics

### Bacteria

Bacteria are classified into Gram-positive and Gram-negative strains. The inner or cytoplasmic membranes of both groups of bacteria are similar; while the outer cell envelopes are significantly different, which explains their tolerance and susceptibility to antimicrobials. Such differences account for the need to develop distinctive strategies for their eradication [9, 10].

#### Gram-negative bacteria envelope

The cell envelope of Gram-negative bacteria consists of three layers: the outer membrane, peptidoglycan cell wall, and cytoplasmic or inner membrane [11]. The outer membrane is a distinctive feature of Gram-negative bacteria because this layer is absent in Gram-positive bacteria. It consists of lipopolysaccharide (LPS) in the outer leaflet and phospholipids in the inner leaflet [11], which acts as a selective impermeable barrier and protects the cell against external toxic threats.

LPS is composed of three structural domains: a hydrophobic lipid section (lipid A), a hydrophilic core oligosaccharide, and a repeating hydrophilic O-antigenic oligosaccharide side chain that contributes to the cell’s pathogenicity [12, 13]. The LPS molecules form a very effective selective impermeable barrier for hydrophobic molecules.

The proteins of the outer membrane are divided into two classes: lipoproteins and β-barrel proteins [14]. The lipoproteins are attached to the inner leaflet of the outer membrane; while the β-barrel proteins are hydrophobic transmembrane proteins [15]. Some β-barrel proteins function as passive diffusion channels such as porins that limit the diffusion of hydrophilic molecules larger than 600 g/mol and render Gram-negative bacteria innately resistant to many antimicrobial compounds [16]. Because of the presence of phosphates and carboxylates in LPS, the outer membrane of Gram-negative bacteria is negatively charged [17] and the charges are higher than those of Gram-positive bacteria [18]. This electrostatic region serves as a primary barrier to most hydrophobic antibiotics, resulting in low permeability.

The peptidoglycan cell wall is made up of repeating units of the disaccharide, *N*-acetyl glucosamine (NAG)-*N*-acetyl muramic acid (NAM), cross-linked by pentapeptide side chains [19]. The cell wall of Gram-negative bacteria consists of a thin peptidoglycan layer (20–50 nm thick) for maintaining the shape of the bacterial cell [11]. Unlike Gram-positive bacteria, the cell wall of Gram-negative bacteria lacks teichoic acid.

**Fig. 2:**
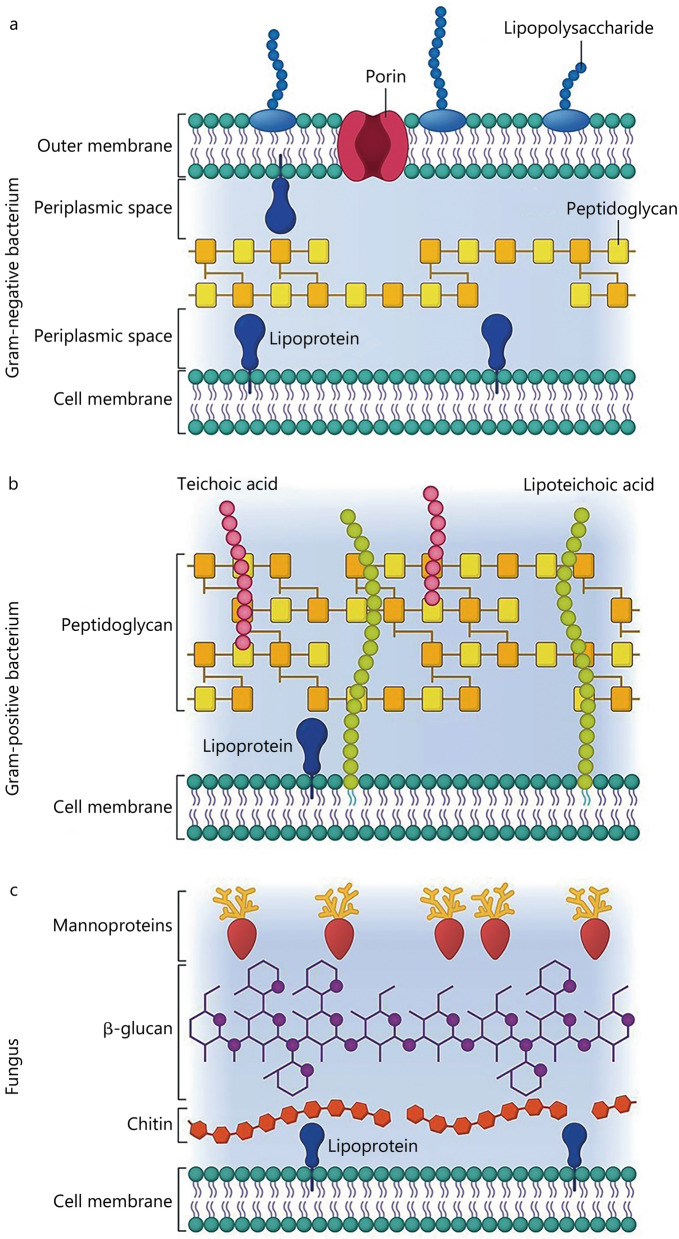
The membrane structure of Gram-negative bacterium (**a**), Gram-positive bacterium (**b**) and fungus (**c**). The cell membranes of both types of bacteria are similar. The Gram-positive bacterium has a thick peptidoglycan layer surrounding the cell membrane. In contrast, the peptidoglycan layer in Gram-negative bacterium is thinner with an additional outer membrane. Fungus cells have an outer cell wall comprising chitin, β-glucan, and mannoproteins

The inner membrane is composed of 40% phospholipids and 60% proteins with a hydrophilic head and a hydrophobic region that makes up the tail part of the structure. The hydrophobic membrane functions as a barrier to regulate the movement of substances in and out of the bacterium. Lying between the two concentric membrane layers is an aqueous cellular compartment called periplasm (Fig. 2a), which acts as a reservoir for virulence factors and sequesters potentially harmful degradative enzymes [11].

#### Gram-positive bacteria envelope

Compared with the cell envelope of Gram-negative bacteria, the protective outer membrane is absent in Gram-positive bacteria. The peptidoglycan layer is also much thicker (15–100 nm) (Fig. 2b) [20]. The Gram-positive bacterial cell wall also consists of long anionic polymers known as teichoic acids. Teichoic acids may be divided into two types: lipoteichoic acids and teichoic wall acids. The lipoteichoic acids are anchored via lipid domains to the cytoplasmic membrane; while the teichoic wall acids are covalently bound to the peptidoglycan layer [21]. The peptidoglycan, lipoteichoic acids, and teichoic wall acids together make up a polyanionic layer that contributes to the structure and function of the cell envelope. The latter has an overall negative charge due to the presence of phosphodiester bonds between the teichoic acid monomers [22]. Some Gram-negative and Gram-positive bacteria produce capsules (or loosely-attached slime layers) on their surfaces [23]. They play prominent roles such as protection against desiccation, phagocytosis by neutrophils or macrophages, phage attack, antibiotics or toxic compounds, osmotic stress, as well as cell recognition [24].

### Fungi

Fungi are eukaryotic organisms that exist in two forms: filamentous or hyphal form (mold), and single-celled or budding form (yeast). They are nucleated and possess distinctively different cell walls from bacteria and viruses. The fungal cell wall makes up 40% of the total cell volume, with thickness ranging from 0.1 to 1.0 μm. It consists of chitin, extensively crosslinked polysaccharides (mainly glucans), and glycoproteins (Fig. 2c) [25]. Glucan is the most important structural component of the fungal cell wall, making up 50–60% of the total cell wall by dry weight [26]. Chitin is a minor component of the fungal cell wall. It is composed of long chains of β-1,4-linked *N*-acetylglucosamine (NAG). Chitin makes up only 1–2 wt% of the yeast cell wall and up to 10–20 wt% of the cell wall of filamentous fungi. It is covalently linked to β-1,3-D-glucan. Interwoven between the chitin and glucan components are proteins, which comprise 30–50% of the dry weight of the fungal wall in yeast and 20–30% of the dry weight of the cell wall of filamentous fungi. These proteins are modified with *O*- or *N*-linked oligosaccharides. The fungal cell wall has many functions, including providing cell rigidity and shape, metabolism, ion exchange, as well as interactions with host defense mechanisms [27].

## Bioengineered nanomaterials for selective microbe killing

### Bacteria-targeting nanomaterials

**Fig. 3:**
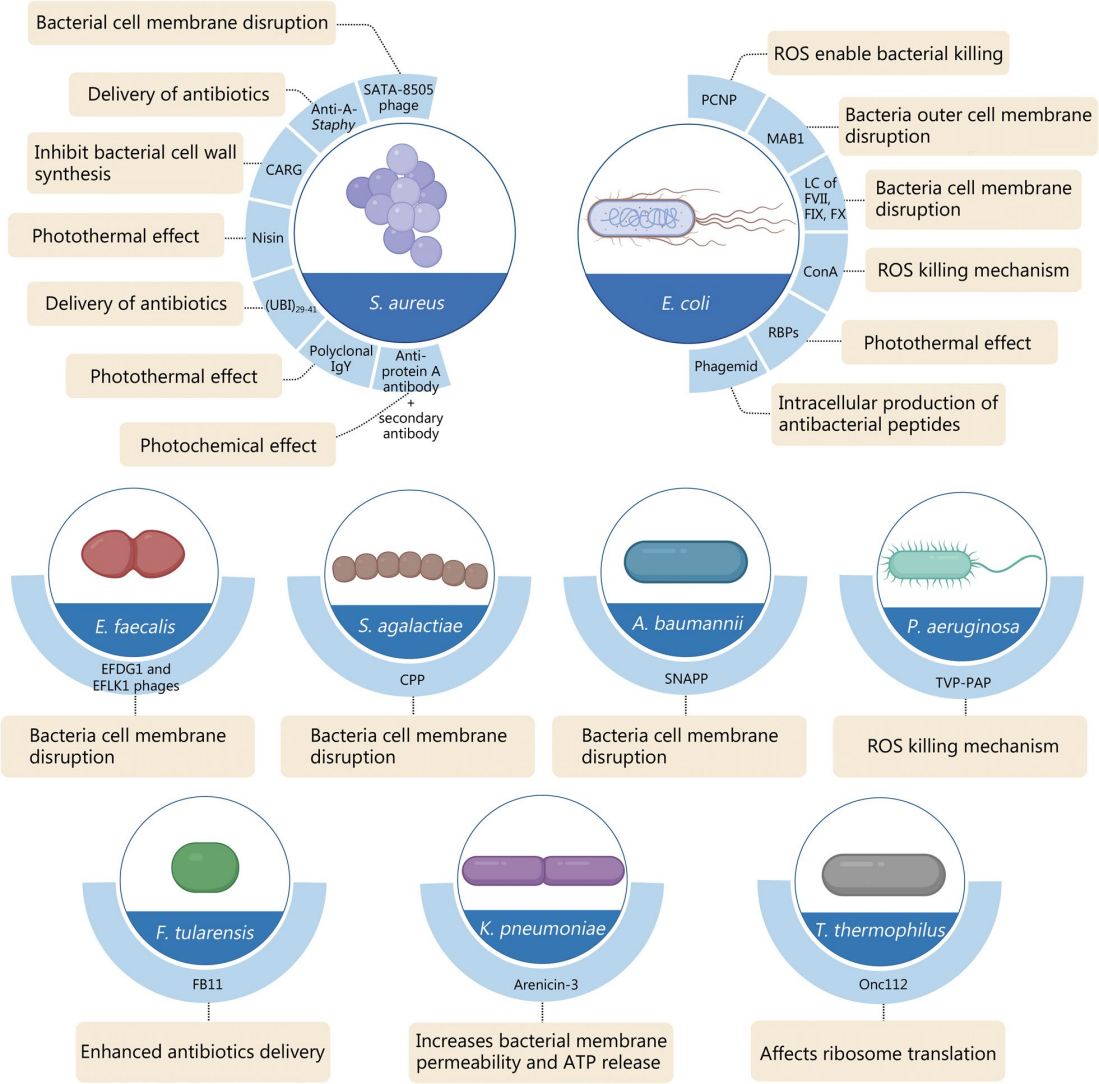
Summary of targeting nanomaterials for selective killing of bacteria. Different specific targeting sites are listed for effective selectivity. *A. baumannii Acinetobacter baumannii*, Anti-A-*Staphy*, Anti-protein A-*Staphylococcus*, *E. coli Escherichia coli*, *E. faecalis Enterococcus faecalis*, *F. tularensis Francisella tularensis*, *K. pneumoniae Klebsiella pneumoniae*, *P. aeruginosa Pseudomonas aeruginosa*, *S. agalactiae Streptococcus agalactiae*, *S. aureus Staphylococcus aureus*, *T. thermophilus Thermus thermophilus*, SNAPP structurally nanoengineered antimicrobial peptide polymer, ATP adenosine triphosphate, Onc112 a proline-rich antimicrobial peptide, TVP-PAP a new type of antimicrobial bioconjugate, CPP cell-penetrating peptide, EFDG1 an anti-*E. faecalis* phage, ROS reactive oxygen species, MAB1 methanoculleus bourgensis strain, RBPs phage-displayed receptor-binding proteins, ConA Concanavalin A, SATA-8505 *Staphylococcus aureus* bacteriophage, FB11 F. tularensis live vaccine strain lipopolysaccharide-specific mouse antibody, EFLK1 anti-*E. faecalis* phage, CARG cyclic 9-amino-acid peptide CARGGLKSC, PCNP phthalocyanine entrapped nanoparticles, LC of FVII, FIX, FX light chain of coagulation factors VII, IX, X

Versatile nanomaterials have emerged over the last decade that demonstrate excellent antimicrobial performance through their intrinsic toxicity or antibiotic delivery capability [28]. However, examples of successful clinical translation of these nanomaterials for treating infectious diseases are scanty. Two silver nanoparticle-based antimicrobial agents, Silvasorb® and NanoAgCVC, are involved in Phase III and Phase IV clinical trials, respectively. These materials possess broad-spectrum bactericidal activity against the skin and central venous catheter infections [29, 30]. As efficient antibiotic delivery promotes bacterial killing, two Phase III clinical trials have been initiated that incorporated commercial antibiotics within liposomes. Although these nano-formulations are bactericidal against Gram-negative bacteria, their selectivity toward specific bacterial strains and their targeting efficacy are unclear [31]. Compared to the direct use of conventional antibiotics in the clinic, the use of nanomaterials could significantly improve the bioavailability and antibacterial potency due to the enhanced loading and sustained release of antibiotics in nano-formulations. Even though in most infection scenarios, antibiotics are empirically prescribed based on the clinical manifestation before identifying the specific strains of the pathogen, the use of nanomaterials could promote the delivery efficiency of antibiotics with a significantly reduced dose burden. Moreover, because microbes and mammalian cells co-exist in complex bacterial infections, direct application of antibacterial agents to the body may result in low therapeutic efficacy, toxicity and inflammation, and unwarranted side effects [32]. Active targeting of nanomaterials to pathogens, especially for the recognition of specific bacterial strains, is highly desirable for clinical applications but remains extremely underexplored [33]. Because of the difference in cell wall characteristics between Gram-positive and Gram-negative bacteria, and also specific strains under each category, there emerged several strategies to allow the recognition of specific pathogens. Among them, case studies of nanomaterials functionalized with targeting ligands for enhanced antibacterial performance are summarized in the present section, while the use of small molecules for bacterial targeting studies were not included here. In general, antimicrobial peptides, antibody/proteins, or bacteriophages are used as targeting ligands to orchestrate bacteria-specific killing. The rational design of antimicrobial peptides or polymers produces selective toxicity against bacteria [34, 35]. The specifications of targeting ligands and the corresponding antibacterial approach mediated by these nanoparticles are summarized in Fig. 3. The typical Gram-positive strain of *Staphylococcus aureus* (*S. aureus*) and Gram-negative strain of *Escherichia coli* (*E. coli*) is the most widely investigated bacteria. Several targeting sites have been identified to enable specific recognition of these bacterial strains. Versatile targeting antibacterial strategies have emerged, such as targeted delivery of antibiotics, cell wall rupture, reactive oxygen species (ROS) denaturation, and photothermal therapy. Targeting sites and associated antibacterial therapies toward other strains are also summarized in Fig. 3 and discussed in detail in the following subsections, as categorized by the targeting strains.

#### Selective killing of Gram-positive bacteria

*S. aureus* is one of the most common Gram-positive pathogens that cause invasive infections. In particular, methicillin-resistant *S. aureus* (MRSA) has attracted intensive research interest because of its threat to public health [36]. Various approaches have been reported for the selective killing of *S. aureus* [37, 38]. Among those approaches, short peptides that can specifically bind to the pathogen have been used extensively as targeting ligands to augment antibacterial performance. The design of targeting peptides may be achieved through in vivo screening of phage display peptide libraries [39]. With the use of a high throughput sequencing and consensus motif analysis, dominant shared peptide sequences on recovered phages in a *S. aureus*-induced pneumonia model were identified. Based on this information, a cyclic 9-amino-acid peptide CARGGLKSC (CARG) has been synthesized as the pathogen-targeting ligand and conjugated on porous silicon nanoparticles for enhanced delivery of vancomycin [39]. This phage-based binding site sequencing strategy is of great significance in the clinic. Given the high complexity and unawareness of pathogen details in an infection, quick screening of phage binding site sequence allows more specific treatment of infectious diseases through active targeting. The CARG targeting peptide shows potent in vitro binding specificity to *S. aureus*, including MSRA, but not *Pseudomonas* bacterial species. This enables the targeting nanoparticles to accumulate selectively in *S. aureus*-infection sites of mice but not in non-infected tissue or *Pseudomonas*-infected sites. The in vivo animal study further demonstrated the excellent antibacterial property of vancomycin delivered by the targeted nanoparticles, which is about tenfold more effective than free vancomycin in suppressing *Staphylococcal* infections. A similar approach has been reported for the selective delivery of gentamicin to *S. aureus* via mesoporous silica nanoparticles that are labeled with a human antimicrobial peptide fragment, ubiquicidin_29–41_, as the targeting ligand [40].

Apart from its function as an antibiotic, vancomycin is also a glycopeptide that may be used as a targeting ligand to specifically integrate with the *d-Ala-d-Ala* terminus of Gram-positive bacterial cell wall peptidoglycan [41]. Selective killing of MRSA has been developed based on vancomycin-loaded gold nanostars. In this system, the vancomycin acts as both the targeting ligand and the antibiotic, with the gold nanoparticles generating heat under near-infrared irradiation to promote antibacterial performance. This targeted chemo/photothermal antibacterial therapy is highly potent in combating MRSA infection, with minimal toxicity and inflammatory consequences [42]. Similarly, the lantibiotic nisin, a bacteria-derived antimicrobial peptide, specifically interacts with the lipid II unit on the cell wall of Gram-positive bacteria such as *S. aureus* [43]. By harnessing its property as a pathogen-specific binding ligand, nisin was modified on the surface of Janus micromotors (i.e., self-propelled micro and nanoscale devices that combine different properties within a single entity) consisting of graphene oxide/Pt nanoparticles/Fe_2_O_3_ [44]. Propelled by catalytic hydrogen peroxide decomposition and magnetic actuation, the micromotors demonstrated a twofold increase in the killing of *S. aureus*, compared to the free peptide and static counterparts. The micromotors demonstrated negligible killing of Gram-negative *E. coli* [44]. The highly precise pathogen specificity and controllable mobility endow the targeting micro/nanomotors with broad applications in treating many infectious diseases using novel approaches.

Much research has been focused on the design of targeting strategies against extracellular bacteria that are present in an infection site or biological fluid. However, the problem of efficiently eliminating intracellular bacteria remains a humongous challenge. Cell-penetrating peptides (CRPs) possess similar physiochemical characteristics as antimicrobial peptides. Although they have been used experimentally for the elimination of intracellular bacteria, CRPs generally have a broad antibacterial spectrum [45]. A cell-penetrating selective antimicrobial peptide has been developed by combining a hydrophobic peptide pheromone of *Streptococcus agalactiae* (*S. agalactiae*) and a cationic CRP [46]. The bacteria-selective peptide acts through bacterial membrane disruption. The platform selectively killed *S. agalactiae* and not other Gram-negative bacteria strains. Apart from combining with the *S. agalactiae* pheromone, the CRP may also be directly conjugated with kanamycin via a reducible linker (P14KanS). This enabled a significant reduction in *Salmonella* levels in an in vivo *Caenorhabditis elegans* model [47].

Monoclonal antibodies are examples of another category of targeting ligands. They have been employed in cancer therapeutics, immunotherapies, and anti-viral therapeutics [48, 49]. However, the use of monoclonal antibodies for selective bacterial killing is limited [50]. By targeting specific virulence proteins on the bacterial surface, these antibodies bestow pathogen-specificity with low cytotoxicity, thus preventing bacterial resistance. In a recent study, a *S. aureus*-targeting antibody, anti-protein A (anti-*Staph*) was used to functionalize poly(d,l-lactic-co-glycolic acid) nanoparticles to enhance the delivery efficacy of rifampicin. This nano-antibiotic showed selective toxicity to *S. aureus* and achieved excellent results in eradicating the planktonic and biofilm versions of the bacterium in vitro. This enabled a significant reduction in *Salmonella* levels in an in vivo *Caenorhabditis elegans* model [51]. Because of antibacterial resistance created by inadvertent antibiotic delivery, physical methods for bacterial eradication have attracted increasing levels of attention. Antibodies that specifically bind with *S. aureus* have been conjugated with porous silica and gold nanoparticles. These assemblies generate a large amount of heat during near-infrared irradiation. A tenfold increase in the bactericidal efficacy of *S. aureus* has been achieved, compared with their effects on *E. coli* [52].

Biodegradable polymer materials based on cationic polyaspartamide derivatives with different lengths of side chains were synthesized through ring-opening polymerization of β-benzyl-l-aspartate *N*-carboxy anhydride [53]. This was followed by an aminolysis reaction and subsequent methylation reaction to produce an antibacterial agent against MRSA. The cationic quaternary ammonium groups contribute to the insertion of the cationic polymer into the negatively-charged bacterial membranes. This resulted in membrane lysis, leakage of bacterial content, and ultimately, death of the pathogens. Apart from MRSA eradication, the biodegradable polymer also possessed alterable antibacterial potency because of its cleavable backbone. This attribute helped to minimize microbial resistance and mitigate drug accumulation. The robust antibacterial system was successfully used to support MRSA-infected wound healing in vivo. After 7 d, the cationic polymer demonstrated about 95% MRSA-killing efficacy [53].

Recently, a phage-guided targeting strategy has been developed for phage therapy to enable sensitive monitoring of the infection as well as selective bacteria eradication [54]. It has been over a century since bacteriophages have been used to treat bacterial infections. This strategy was reincarnated in the past 15 years due to its promising application in suppressing antimicrobial resistance [55]. Compared with the peptides and antibodies mentioned previously, bacteriophages have precise targeting capability on infectious bacteria. They offer a safe and effective approach to dealing with complex infections, especially those caused by multidrug-resistant bacteria [56]. Mediated by receptor-binding proteins, bacteriophages such as P2 and TP901-1 can specifically recognize the Gram-positive bacterium *Lactococcal lactis* [57]. The peptidoglycan cell wall on the outermost layer of Gram-positive bacteria also serves as the targeting site for the binding of bacteriophages. Phage-mimicking nanoparticles and the use of nanoparticles for triggering the release of bacteriophage endolysin have been reported as strategies to promote antibacterial efficacy [58, 59].

#### Selective killing of Gram-negative bacteria

Antibiotic-resistant bacterial infections caused by Gram-negative organisms have become a global health threat [60]. Unlike Gram-positive bacteria, Gram-negative bacteria have an outer membrane layer outside the peptidoglycan layer. This phospholipid membrane provides an extra barrier that prevents certain antibiotics from diffusing inward. The outer membrane protects the bacterium against cell wall lysis, increasing the likelihood of resistance. To address this issue, conventional antimicrobial peptides will need to be re-designed to fit the new target. “Structurally nanoengineered antimicrobial peptide polymers” (SNAPPs) have been developed to satisfy this goal. This is a new class of antimicrobial agents with high sensitivity and specificity to Gram-negative bacteria [61]. Unlike conventional peptide-assembled antimicrobial macromolecules, SNAPPs were designed with a polymer core and repeating peptide units of lysine and valine* N*-carboxy anhydride on their surfaces. They target Gram-negative bacteria via a multi-modal antibacterial mechanism. This peptide-decorated nanoagent was highly effective in combating multidrug-resistant *Acinetobacter baumannii* (*A. baumannii*) infection in mice, with excellent clinical translation potential.

Apart from bacterial surface ligand recognition, antimicrobial peptides that function inside particular strains of bacteria are also useful for efficient bacterial killing. A proline-rich antimicrobial peptide (Onc112) binds specifically with the inner membrane protein SbmA in Gram-negative bacteria to facilitate its transportation into the cytoplasm [62]. The Onc112 peptide exerts its antimicrobial action by affecting the initiation of ribosome translation via the formation of unstable initiation complexes. This prevents the affected ribosome from entering the translation stage.

Chemodynamic and photodynamic therapies that generate high concentrations of ROS have been used for eradicating bacteria. However, these therapies usually have a broad antimicrobial spectrum [63]. The LPS present on the Gram-negative bacteria’s outer membrane typically prevent photosensitizers from binding to the bacteria, thereby protecting the cells from chemical attack. To solve this problem, a polycationic peptide, KRKKRKKRK (CPNP), that competitively displaces divalent cations from LPS in Gram-negative bacteria was conjugated to the surface of L-type cysteine (L-Cys)-decorated cadmium telluride nanoparticles (CdTeNPs) [64]. This nanoscopic antibacterial agent has been used for fluorescence imaging-guided antibacterial therapy for selective adhesion to Gram-negative bacteria such as *E. coli* and *Pseudomonas aeruginosa* (*P. aeruginosa*) to augment ROS production for eradication of these bacteria.

Other classes of biological substances are also capable of selective conjugation to Gram-negative bacteria by targeting their surface LPS [65]. As a class of glycoconjugates, LPS consist of a hydrophobic lipid domain (lipid A) anchored to the bacterial outer membrane, a polysaccharide consisting of repeating oligosaccharide units of 2–8 sugar residues (O-antigen), and an oligosaccharide chain (core-OS) linking the lipid A and the O-antigen [66]. New antimicrobial agents have been produced that bind specifically to these LPS components for the selective killing of Gram-negative bacteria. For example, Concanavalin A, a lectin protein, has been used to target Gram-negative bacteria because of its specific binding to the mannosyl and glucosyl residues of LPS. The photosensitizer rose bengal showed a 117-fold improvement in antibacterial performance after conjugation to Concanavalin A. This was achieved by augmenting the local oxidative stress that damages the cell membrane of *E. coli* [67]. Three mammalian coagulation factors (VII, IX, and X) that initiate clotting, were recently found to possess intrinsic antimicrobial activity against Gram-negative bacteria via hydrolysis of LPS [68]. Both in vitro and in vivo studies demonstrated that these coagulation factors were effective in controlling drug-resistant Gram-negative bacterial infections caused by *P. aeruginosa* and *A. baumannii*.

Antibodies that possess potent binding specificity have also been employed for pathogen targeting. Instead of directly interacting and interfering with the bacterial surface component, the antibody-antigen interaction is used as an “intelligent” nanovalve for specific pathogen-triggered release of antimicrobial agents. For example, the monoclonal antibody against *Francisella tularensis* (*F. tularensis*) LPS, anti-*F. tularensis* LPS antibody (FB11), binds specifically to a tetrasaccharide derived from the O-antigen of the bacterium’s LPS. Such a property was utilized for the development of an antimicrobial agent with selective toxicity against *F. tularensis*, a Gram-negative coccobacillus that causes tularemia (rabbit fever), the pneumonic form of which is often lethal without treatment. Mesoporous silica nanoparticles were first loaded with an antibiotic. This was followed by surface modification with a derivative of the O-antigen from *F. tularensis* LPS. The drug-loaded mesopores were subsequently capped with the large FB11 antibody to block the pore opening. This helped to reduce the premature release of the cargo drug molecules before reaching the specific pathogen [69]. Upon reaching the bacterial surface, the FB11 antibody bound effectively with the native LPS on the outer membrane of *F. tularensis*. Interaction of the antibody with the antigen opened the pores in the mesoporous silica nanoparticles and released the antibiotic for the targeted killing of *F. tularensis*. The high selectivity of the targeted treatment reduced side effects and was associated with a lower risk of resistance when compared to the use of conventional broad-spectrum antibiotics. Apart from the LPS domain, other components from Gram-negative bacteria may also be targeted to achieve species-specific killing. For example, a monoclonal antibody that targets an extracellular epitope of *E. coli*’s β-barrel assembly machinery subunit (BamA) was used experimentally to promote antibacterial activity via inhibition of β-barrel protein folding and disruption of the integrity of the bacterial outer membrane [70].

Compared with Gram-positive bacteria, phage-guided antibacterial strategies are more commonly used for treating diseases associated with Gram-negative bacterial infections. There is a clinical study on phage therapy-based infection treatment [71]. However, bacteriophages typically show high bacterial specificity but relatively low antibacterial activity. To solve this dilemma, novel strategies have been developed that mimic the antibacterial activity of bacteriophages. For example, the aggregation-induced emission (AIE) concept was integrated with phage therapy by engineering bacteriophages that are equipped with luminogens that bear AIE characteristics (i.e., AIEgens) [72]. The AIEgen TVP-S (an AIE compound) allowed real-time monitoring of specific bacterium-phage interactions. An in vivo wound model was established to evaluate the antibacterial capability of the AIEgen-phage bioconjugates, particularly in treating multidrug-resistant *P. aeruginosa* infection.

Photothermal therapy has also been integrated with phage therapy. In this strategy, chimeric phages with strong pathogen specificity were conjugated with gold nanorods, a typical photothermal nanoagent. The high temperature generated by the photothermal effect derived from the gold nanorods induced bacterial ablation. The phages were destroyed at the same time to prevent overdosing and reduce potential side effects [73]. Because conventional phages are lysogenic, they disrupt bacterial cell membranes and release a large number of endotoxins. This may result in detrimental side effects such as inflammation, sepsis, and even death. To resolve this issue, a modular bacterial phagemid system was engineered which expressed nonlytic antimicrobial peptides and toxin proteins to treat bacterial infection [74]. Phage-based antibacterial approaches allow species-specific pathogen killing with reduced prevalence of antibiotic resistance. These approaches demonstrate clinical translation potential for the treatment of chronic infections.

Other forms of surface modification such as the addition of branched poly(ethylene imine) to silver nanoclusters have been used to selectively kill multidrug resistance bacteria without creating biocompatibility issues [75]. Selectivity of the silver nanoparticles was further improved by conjugation of the cell wall binding domain of the defined pathogen, with minimal effect on microbiota [76]. For instance, the wall binding domain from *Bacillus anthracis* (*B. anthracis*) was linked to Ag nanoparticles. Such a hybrid combination was selectively bound to *B. anthracis* and subsequently killed this bacterial strain [76]. The CBD^BA^ from *B. anthracis* selectively bind to *B. anthracis* in a mixture with *Bacillus subtilis*, as well as in a mixture with *S. aureus* [76]. Such a biotic-abiotic hybrid was capable of recognizing its specific target cells in a bacteria mixture [76]. This new biologically-assisted hybrid strategy has the potential to selectively eradicate pathogenic bacteria, with minimal impact on the normal microflora.

**Fig. 4:**
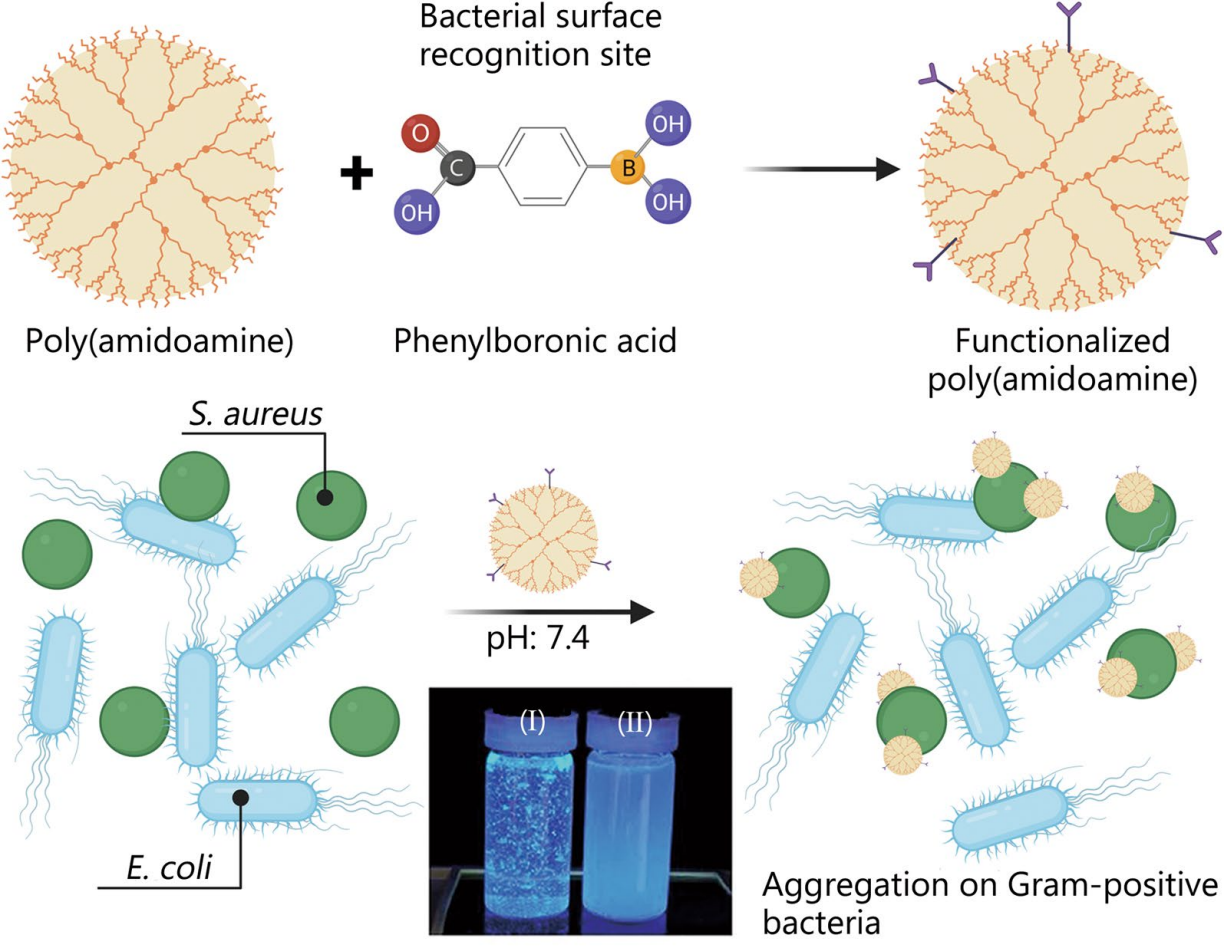
Selective toxicity using functionalized materials that attach to specific microbes. Schematic of the fabrication of boronic acid-functionalized poly(amidoamine) dendrimer to target Gram-positive bacteria. *Staphylococcus aureus* (*S. aureus*) (I) and *Escherichia coli* (*E. coli*) (II) are shown in the photograph. Reprinted from American Chemical Society from ref[77] under open access license

Phenylboronic acid is capable of discriminating bacterial surfaces. This desirable property provides the incentive for the synthesis of boronic acid-functionalized poly(amidoamine) dendrimer (Fig. 4) [77]. In a mixture of Gram-positive *S. auerus* and Gram-negative *E. coli* in physiological pH, this material aggregated on the *S. auerus* only. This is attributed to the presence of phenylboronic acid for bacterial surface recognition.

A cell membrane coating endows a nanoparticle core with prolonged systemic circulation and cell-specific targeting [78, 79]. In light of this, a bacterial membrane-coated nanosystem was prepared to enhance bacterial targeting and uptake of rifampicin, an antibiotic that is not effective against Gram-negative *E. coli* [80]. The ineffectiveness of the antibiotic is attributed to the double-membrane structure of *E. coli*, which inhibits the hydrophobic antibiotic from crossing the membrane barrier [81]. The nanosystem was constructed by coating the surface of rifampicin-loaded mesoporous silica nanoparticles with outer membrane vesicles isolated from *E. coli*. Because of the homologous targeting function of the outer membrane vesicles, the *E. coli*-derived shell significantly improved the uptake of the bacterial membrane-coated nanostructures by *E. coli* [82]. This mode of selective targeting was not observed in the Gram-positive *S. aureus.* These facts were confirmed by the flow cytometry results and confocal laser scanning microscopy (CLSM) images. The enhanced uptake of rifampicin conferred the functionalized nanosilica with superior antibacterial activity against *E. coli*. A single treatment with the bacterial membrane-camouflaged system improved the survival rate of the infected mice and reduced bacterial load in the intraperitoneal fluid and organs in a peritonitis mouse model [82]. This example demonstrated how one can design and construct an outer membrane vesicles-coated nanosystem for the treatment of Gram-negative bacterial infections. This innovative biomimetic improves the antimicrobial efficacy of conventional antibiotics.

### Fungi-targeting nanomaterials

The escalating annual increase in fungal infections echoes the urgency of antifungal research [83]. Antifungal compounds have been developed that target the fungal cell wall, especially by inhibiting the synthesis of essential cell wall components such as chitin, β-1,3-D-glucan, or ergosterol [84]. Many of these antifungal agents have their drawbacks in terms of pharmacokinetic characteristics, bioavailability, and safety issues [85]. Nanomaterials such as lipid nanoparticles, liposomes, and polymeric particulates have been reported to facilitate the safe and efficient delivery of anti-fungal compounds [86]. For example, amphotericin B is a commonly used antifungal agent. The liposomal formulation AmBisome is used as a first-line clinic medication for treating fungal infections [87]. To increase the specificity of the liposome-AmB formulation in targeting fungi, a dectin-1 β-glucan binding domain (known as the β-glucan receptor in humans) was conjugated to the liposome surface [88]. As a mammalian innate immune receptor in the plasma membrane of leukocytes, dectin-1 binds strongly to β-glucans on fungal cell walls. Conjugation of this fungal targeting ligand to liposomes resulted in significantly improved therapeutic performance and reduction in the effective dose of amphotericin B.

**Fig. 5:**
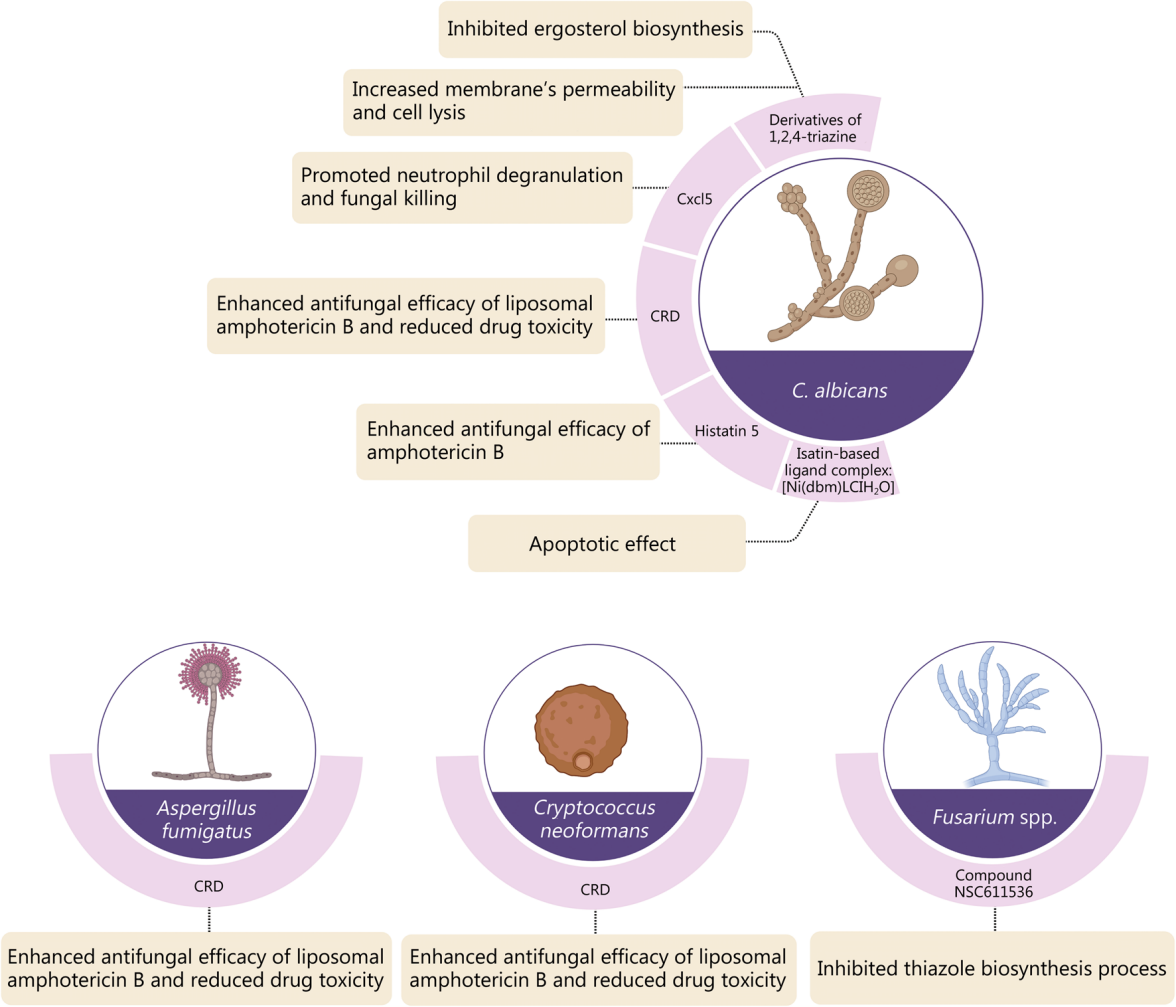
Summary of targeting nanomaterials for selective killing of fungi. Five specific targeting sites for *Candida albicans* (*C. albicans*) and associated antifungal therapies are listed. Targeting strategies for the other three fungi, *Aspergillus fumigatus*, *Cryptococcus neoformans,* and *Fusarium* spp*.* are also shown. CRD chronic respiratory disease, Cxcl5 CXC chemokine ligand 5

Unlike bacteria in which organelles are typically absent, the intracellular components of fungi may also be targeted for the selective antifungal property. Mitochondria have been recognized as the “power factory” in cells that provide the necessary energy for physiologic biochemical reactions. The mitochondrial phosphate carrier protein plays a key role in promoting mitochondrial oxidative phosphorylation through the transport of phosphoric acid to the mitochondria [89]. Accordingly, thiohydantoin ML316 was developed that exhibited fungal-selective inhibition of the mitochondrial phosphate carrier Mir1. This resulted in the highly effective killing of drug-resistant *Candida* species. Another agent targeting *Candida parapsilosis* has also been developed by inhibiting the splicing of group II introns (a tertiary structure of mitochondrial RNA) [90]. Succinate dehydrogenase, being a crucial component of the respiratory enzyme complex, has similarly been utilized as the target for the development of novel agricultural fungicides [91, 92]. Figure 5 represents a summary of targeting nanomaterials for the selective killing of fungi.

There is ample research that utilizes nanoparticles for antifungal applications. Examples include the use of ZnO and TiO_2_ nanoparticles for ROS-augmented pathogen killing [93, 94], as well as the use of chitosan-based nanoparticles for the delivery of fungicides [95, 96]. However, nanomaterials that can selectively recognize specific fungal species, or those that can differentiate the antifungal effect from the antibacterial and cytotoxic effects on mammalian cells are still scanty. There is pressing clinical demand for safe and efficient antifungal agents. The development of innovative strategies for fungal-selective killing has highly-esteemed scientific merits and translation potential.

Azole compounds, particularly fluconazole, are the most commonly used antifungals because they are highly advantageous over other antifungal drugs in terms of cost, safety, oral bioavailability, and the ability to cross the blood–brain barrier. Unfortunately, the repeated use of fluconazole for the treatment of fungal infections has resulted in the emergence of multidrug resistance fungal isolates that exhibit resistance to other azoles, such as itraconazole and voriconazole. Recently, the study of the structure–activity relationship of oxadiazolylthiazole antibiotics unexpectedly led to the identification of ethylenediamine- and propylene diamine-analogs as potential antimycotic novel lead structures. Replacement of the ethylenediamine moiety with the cis-diaminocyclohexyl group significantly enhanced the antifungal activity of corresponding compounds. These compounds showed highly selective broad-spectrum activity against 20 drug-resistant, clinically important fungi, including *Candida species*, *Cryptococcus,* and *Aspergillus fumigatus* strains, without inhibiting the human normal microbiota [97]. Table 1 shows a summary of nanomaterials with microbial selectivity for biomedical applications.

**Table 1:** Representative bioengineered nanomaterials with selectivity toxicity against microbes

Type of materials	Type of microbes	Selectivity	Application	References
Hollow outer membrane vesicles coated with bovine serum albumin	Gram-negative bacteria	*K. pneumoniae*	Infection resulting from carbapenem-resistant *K. pneumoniae* in bladder	[98]
Outer membrane vesicles coated with gold nanoparticles	Gram-negative bacteria	*E. coli*	Antibacterial vaccine	[99]
Chitosan nano-structures coupled with synthetic recombinant antigens	Gram-negative bacteria	*E. coli*	Antibacterial vaccine	[100]
Gold nanoparticles	Gram-negative bacteria	*E. coli*	Antibacterial vaccine	[101]
Oxadiazolylthiazoles	Fungi	*Candida*	Treatment of fungal infections	[97]
Chitosan and its nanoparticles	Fungi	*C. albicans*	Antifungal agent	[95]
TiO_2_ nanoparticles co-doped with silver and nitrogen	Gram-positive bacteria	*B. subtilis*	Antibacterial agent	[94]
WS_2_/ZnO nanohybrid	Fungi	*C. albicans*	Antifungal material	[93]
Bacterial outer membrane‐coated mesoporous silica nanoparticles	Gram-negative bacteria	*E. coli*	Gram-negative bacterial infections	[80]
Silver nanoparticle-cell wall binding domain	Gram-positive bacteria	*B. subtilis*	Antibacterial agent	[76]
ZnO nanoparticles	Gram-positive bacteria	*P. aeruginosa*	Antibacterial agent	[102]

*K. pneumoniae, Klebsiella pneumoniae*; *E. coli, Escherichia coli*; *C. albicans, Candida albicans*; *B. subtilis, Bacillus subtilis*; *P. aeruginosa, Pseudomonas aeruginosa*

## Immune action of antimicrobial materials: immunomodulation and antimicrobial responses

Apart from direct killing the microorganisms by using the aforementioned functional agents, nanomaterials can selectively combat bacterial and fungal infections by instructing the immune system to counterattack the invasion of pathogens. For instance, nanomaterials may be employed as vaccine vectors to deliver antigenic proteins aimed to trigger specific humoral and cellular immune responses against life-threatening multidrug-resistant pathogens. Nanostructures conjugated to bacterial antigenic proteins can selectively induce antibacterial immunity. Metal or chitosan nanoparticle scaffolds were conjugated to antigens derived from the enterohemorrhagic *E. coli* strain; the latter is responsible for the human hemolytic uremic syndrome [100, 101]. Preclinical in vivo administration of these antibacterial formulations induced high serum IgG and mucosal IgA titers. The result is correlated with significant protection against challenges with a specific strain of the enterohemorrhagic *E. coli* bacteria [100, 101]. Reduction in the enterohemorrhagic *E. coli* intestinal colonization is an indicator of the antigen-specific bactericidal properties of the evoked antibodies.

A new promising strategy to fight bacterial infection exploits the possibility of combining synthetic nanoparticles with natural cellular materials to generate biomimetic nanoparticles with the capability to selectively stimulate immune responses toward a specific pathogen. Small gold nanoparticles coated with *E. coli* outer membrane were used to immunize mice. These complexes rapidly induced the maturation of dendritic cells, activation of T cells with interferon-gamma (INF-γ) and interleukin (IL)-17 release, and strong and long-lasting antibody response against bacteria [99].

Recently, outer membrane vesicles derived from Carbapenem-resistant *Klebsiella pneumoniae* (*K. pneumoniae*) were deposited onto bovine serum albumin-coated nanoparticles to obtain nanoparticle-outer-membrane vesicles with enhanced stability and homogenous size. Vaccination with these nanoparticles induced the production of high titers of bacterium-specific antibodies as well as in vivo protection from lethal dose administration of antibiotic-resistant *K. pneumoniae* [98]*.*

**Fig. 6:**
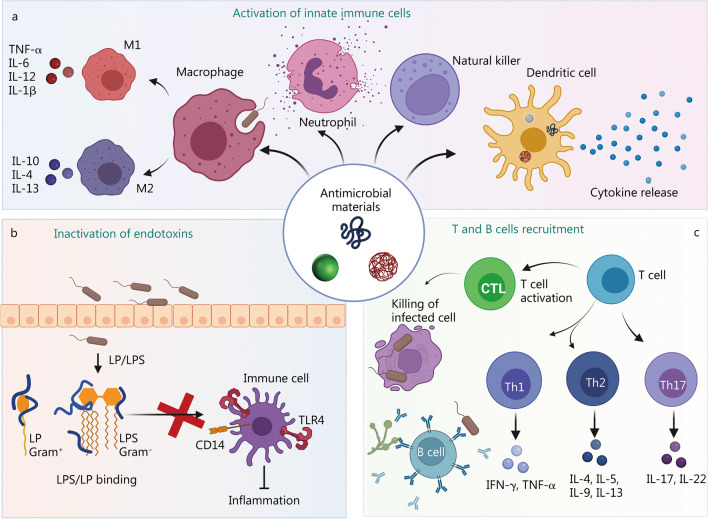
Immune effects of antimicrobial materials. **a** Nanomaterials with selective toxicity toward microorganisms can exert different effects on immune responses. They can recruit macrophages, neutrophils, dendritic cells, and natural killer cells, induce innate immune cell differentiation, promote the release of inflammatory cytokines, as well as kill pathogens. **b** Direct binding of Gram-positive or Gram-negative endotoxins (LPS or LP) by antimicrobial peptides blocks the binding of endotoxins to immune cell receptors like TLR4 or CD14. This results in the prevention of the over-activation of immune cells and the down-regulation of inflammatory responses and sepsis. **c** Bacteria or fungal antigens exposed on nanomaterials can be recognized by adaptive immune cells to activate Th, cytotoxic T cell as well as B cell adaptive immune responses, promoting bacterial clearance via opsonization mediated by complement and/or antibodies produced by B cells or direct killing of infected cells by cytotoxic T lymphocytes (CTLs). IL interleukin, TNF-α tumor necrosis factor-α, IFN-γ interferon-gamma, LPS lipopolysaccharide, LP lipoprotein, TLR4 Toll-like receptor 4, Th T helper

Apart from their direct action on pathogens, some nanomaterials combine selective toxicity properties with immunomodulating characteristics, directing the immune system to fight bacterial infections. Antimicrobial nanostructures can exert their immunomodulatory actions by inducing the recruitment of immune cells to the infection site, as well as augmenting immune responses to expedite microbial elimination [103, 104]. Pathogen-associated molecular patterns (PAMPs) promote the functions of innate immune cells like neutrophils, dendritic cells, macrophages, and natural killer cells (Fig. 6a). These effector cells initiate inflammatory responses via the secretion of soluble mediators such as tumor necrosis factor-α (TNF-α), interferon-γ, as well as pro-inflammatory interleukins such as IL-1β, IL-6, IL-12, and IL-18 [105]. Neutrophils, macrophages, and T cells are attracted by chemokines and other soluble factors to promote bacterial clearance through the induction of neutrophil extracellular traps and macrophage phagocytosis.

The binding of bacterial endotoxins to Toll-like receptors (TLRs) can be prevented by using anti-LPS peptides with high affinity for Gram-negative LPS or Gram-positive lipoproteins, blocking detrimental inflammatory effects (Fig. 6b). Innate immune cell maturation prompts adaptive immune responses inducing activation of T cell subsets with differing functions. These cells promote bacterial clearance by opsonization of the bacteria mediated by complement and antibodies produced by B cells or killing of infected cells by T cytotoxic lymphocytes (Fig. 6c).

Some organic-based materials (e.g., polycarbonate) [106] and inorganic nanostructures (e.g., modified Ag nanospheres [76] and functionalized nanosilica [107]) are capable of simultaneously inducing selective toxicity to specific microorganisms and modulating the host immune system to fight bacterial infections. For example, Ag nanoparticles upregulate IL-1, IL-6, and TNF-α cytokines, as well as the release of IL-1β in primary monocyte culture [108]. This, in turn, activates many signaling pathways linked to innate immunity. Apart from silver, other inorganic antimicrobic materials have been shown to trigger a cascade of immune reactions. For example, the antibacterial properties of zinc oxide nanoparticles are attributed to the production of IL-6, IL-1β, IL-8, and TNF-α in primary human peripheral blood cells [109].

Some antimicrobial materials are formulated to suppress pathogen-associated danger signals. Suppression of these signals induces the release of anti-inflammatory cytokines and inhibits the secretion of pro-inflammatory cytokines. Materials with selective toxicity and intrinsic anti-inflammatory properties are preferred for therapeutic applications. For example, biogenic selenium nanoparticles were exploited against antibiotic‐resistant *P. aeruginosa* and *Candida* spp. without inducing a significant increase in the secretion of pro‐inflammatory and immunostimulatory cytokines or ROS secretion. The safety profile of these antimicrobial selenium nanoparticles has been validated in vivo [110]. Nanocapsules of titanium dioxide containing silver as an antibacterial agent demonstrated potent antimicrobial activity against both *E. coli* and *S. aureus* and even against a multidrug-resistant strain of *S. aureus* without upregulation of IL-6 or co-stimulatory markers in macrophages [111].

Metal-containing antimicrobial agents not only show good biocompatibility with human cells by selectively killing pathogenic bacteria without triggering inflammation, but are also able to turn off the inflammatory processes triggered by the pathogens themselves. As such, silver nanoparticles were developed to fight multidrug-resistant *Salmonella*. The nanoparticles possessed the ability to suppress inflammatory reactions caused by the infection [112]. Similarly, antimicrobial silver-containing silica nanorattles (Ag@SiO_2_) designed for selective toxicity against *E. coli* or *S. aureus* were actively uptaken by dendritic cells without affecting their vitality or impairing immune activation upon simulation of PAMPs [113].

Macrophages also are critical cells in antimicrobial responses. Antibacterial silver nanoparticle-loaded TiO_2_ nanotubes were fabricated for local delivery of Ag ions and antibiotics. These assemblies modulated inflammatory responses and promoted bone regeneration. These effects were achieved by inducing the polarization of macrophages toward an M2 phenotype, instead of an M1 phenotype. The M2 phenotype is associated with the resolution of inflammation and healing, whereas the M1 phenotype is responsible for chronic inflammatory responses [114].

Recent studies showed that nanosilver alters intestinal microbiota composition and exerts intestinal anti-inflammatory effects [115, 116]. In an animal model of ulcerative colitis and Crohn’s disease, treatment with Ag nanoparticles reduced the number of adherent/invasive *E. coli* and *Clostridium perfringens*, and increased the number of beneficial *Lactobacillus* spp.. The Ag nanoparticles also possessed anti-inflammatory activity and suppressed neutrophil recruitment and infiltration. These features resulted in the amelioration of the symptoms of colitis in these experimental models [117]. Collectively, the results demonstrate how materials with selective toxicity can differently modulate the outcome of immune responses.

Synthetic antimicrobial peptides are compounds derived from natural antimicrobial peptides such as cathelicidins, defensins, and dermicines. They possess both antimicrobial activity and anti-inflammatory action [118]. Endotoxins such as LPS or lipoproteins/peptides of the bacterial envelope are strong inductors of TLR-mediated inflammatory responses. The lactoferricin peptide, LF11 (AA 21–32) was modified by coupling of a C12-alkyl group (lauryl-LF11) at the N-terminus. This resulted in the enhancement of the antibacterial action and LPS-induced inflammation inhibition [119]. Similarly, synthetic granulysin-derived peptides simultaneously kill both Gram-negative and Gram-positive bacteria and neutralize the activity of LPS-induced cytokines [120]. Beta-peptide polymer mimicking host defense peptides have the dual functions of reducing the viability of *P. aeruginosa*-established biofilms together with immunomodulation and suppression of LPS-induced pro-inflammatory cytokines [121].

Synthetic anti-LPS peptides (SALPs) represent a powerful approach to fighting bacterial infection by neutralizing LPS-induced inflammation and sepsis. SALPs bind with high-affinity bacterial endotoxins, such as Gram-negative LPS or Gram-positive lipoproteins, to inhibit their binding to TLRs. The synthetic anti-LPS Pep19-2.5 demonstrated the ability to reduce inflammatory cytokine release, downregulate the expression of maturation markers in both human-derived dendritic cells and Langerhans-like cells, and inhibit dendritic cell migration [122]. Pep19-2.5 and its derivatives neutralized LPS and other PAMPs derived from pathogenic bacteria in vivo. Peptide administration in an animal model strongly inhibited TNF-α and IL-6 release and enhanced mice survival after sepsis-inducing toxin administration. The mode of action of these peptides is based on the inhibition of toxin-induced inflammation instead of bacterial killing. SALPs act by sequestering LPS or lipoproteins, both in soluble form or as a constituent of the bacterial cell wall. This resulted in the blocking of their binding to TLR2 and TLR4, as well as preventing the activation of the intracellular signaling cascades mediated by inflammasomes. These activities help to suppress immune-inflammatory over-reactions [123].

Bioactive peptides derived from the iron-binding protein lactoferrin possess potent antifungal, antibacterial and antiviral activities in conjunction with a broad spectrum of immunomodulating proprieties. For example, the lactoferrin (1–11) peptide possessed antifungal activity against *C. albicans* and *A. fumigatus.* The peptide was capable of inducing monocyte-macrophage differentiation, with enhanced phagocytosis of the fungal pathogens. In addition, this peptide induced the maturation of dendritic cells upregulated the expression of human leukocyte antigens class II, and stimulated the production of ROS, IL-6, and IL-10. The differentiated dendritic cells, in turn, induced T helper cell polarization towards Th17 cells to enhance host antifungal responses [124, 125].

Another class of lactoferrin peptides, the lactoferricins, also possess an anti-inflammatory effect. The lactoferricins are capable of neutralizing LPS and other PAMPs such as CpG sequences of microbial origin [126]. Upon reaching the nucleus of host cells, lactoferricin peptides act as antagonists of LPS-activated nuclear factor (NF)-κB to downregulate the secretion of pro-inflammatory cytokines [127].

Antimicrobial polymers represent a promising alternative to synthetic antimicrobial peptides, having higher protease stability and lower production costs. Along with potent antimicrobial properties, antimicrobial polymers also have immunomodulating properties. For example, ultra-short triazine-based amphipathic polymers not only possess antibacterial activities against drug-resistant pathogens, but they also possess anti-inflammatory properties by inhibiting TNF-α and reducing mast cell infiltration and pro-inflammatory cytokine expression in a BALB/c model of atopic dermatitis caused by bacterial colonization [128]. Similarly, synthetic antimicrobial β-peptide polymers have multiple functions. They inhibit the formation of *S. aureus* and *P. aeruginosa* biofilms, promote the release of anti-inflammatory cytokines and chemokines, and the production of pro-inflammatory IL-1β and TNF-α that are induced by bacterial endotoxins. These responses resemble the ones obtained in vivo with natural host defense peptides like α-defensins and the human cathelicidin peptide LL-37 [121].

Immobilization of bioactive peptides on the surface of nanostructures is a potent approach to potentiate the inhibition of bacterial adhesion and block biofilm formation. The antimicrobial KR-12 peptide was covalently immobilized on a titanium surface via the use of a PEGylated spacer. The KR-12 peptide demonstrated improved antibacterial and anti-endotoxin activities, as well as anti-inflammatory capability. They are capable of blocking macrophage activation and reducing-IL-1β and TNF-α release [129]. Some of the antimicrobial materials and their effects on the immune system are summarized in Table 2.

**Table 2:** Some antimicrobial materials and their effects on immune system

Type of material	Function	Selectivity	Type of effect on immune response	References
*E. coli* adherence proteins encapsulated in chitosan NPs	Antibacterial vaccine	Enterohemorrhagic *E. coli* O157:H7	Humoral and mucosal immune responses	[100]
*E. coli* outer membrane proteins immobilized on gold NPs	Antibacterial vaccine	Enterohemorrhagic *E. coli* O157:H7	Humoral and mucosal immune responses	[101]
Gold NPs coated with *E. coli* OMVs	Antibacterial vaccine	*E. coli*	Dendritic cell maturation, Antibody response, Th1 and Th17 T cell responses	[99]
BSA-coated NPs with *K. pneumoniae* OMVs	Antibacterial vaccine	*K. pneumoniae*	Antibody response, T cell response	[98]
Silver NPs	Antimicrobial	*S. Enteritidis*	IL-1, IL-6 and TNF-α cytokine release by macrophages	[107] [111]
3-aminopropyltriethoxysilane ZnO NPs	Antimicrobial	Gram-negative bacteria	IL-6, IL-1β, IL-8 and TNF-α cytokine release	[108]
Silver-containing nanocapsules of titanium dioxide	Antimicrobial	*E. coli*, *S. aureus*	Macrophage phagocytosis enhancement	[110]
Silver-containing silica nanorattles	Antimicrobial	*E. coli*, *S. aureus*	DCs phagocytosis enhancement	[112]
Silver NPs-loaded TiO_2_ nanotubes	Antimicrobial	*-*	Macrophage polarization towards the M2 phenotype	[113]
β-peptide polymer (20:80 Bu:DM)	Antimicrobial	*P. aeruginosa*	Anti-inflammatory activity, suppression of LPS-induced TNF-α. Anti-inflammatory IL-1RA induction. Monocytes/macrophages recruitment	[118]
Peptide19-2.5 and Pep19-4LF derivative	Anti-LPS	Gram-negative and Gram-positive	Anti-inflammatory activity. IL-6 reduction, expression of maturation markers and DC migration inhibition	[119]
Lactoferrin (1–11) peptide	Antimicrobial anti-LPS	*S. aureus, C. albicans, A. fumigatus*	Enhanced phagocytosis and DC maturation, IL-6, IL-10 cytokine release, Th17 T cell polarization	[121]
Lactoferricin peptides	Antimicrobial, anti-LPS	*-*	Anti-inflammatory activity, TNF-α and IL-6 reduction	[124]
Short triazine-based amphipathic polymers	Antimicrobial	*P. aeruginosa*	Anti-inflammatory activity, reduce mast cell infiltration and TGF-β, TNF-α, iNOS, COX-2 levels. Regulates the Th1/Th2 and serum IgE and IgG2a levels	[125]
KR-12 peptide immobilized on titanium surfaces	Antimicrobial	*S. epidermidis*	Anti-inflammatory activity, modulates macrophage activation and IL-1β and TNF-α release	[126]
Lauryl-LF11 peptide	Antimicrobial, anti-LPS	*S. enterica*	Anti-inflammatory activity. Reduction of LPS-induced TNF-α release in human mononuclear cells	[127]
Granulysin-derived peptide Gran1	Antimicrobial, anti-LPS	*M. tuberculosis*	Macrophage phagocytosis enhancement	[128]

*NP* nanoparticle, *OMV* outer membrane vescicle, *BSA* bovine serum albumin, *DC* dendritic cells, *Th* T helper, *IL* interleukin, *TNF* tumor necrosis factor, *LPS* lipopolysaccharide, *E. coli Escherichia coli*, *K. pneumoniae Klebsiella pneumoniae*, *S. Enteritidis Salmonella Enteritidis*, *S. aureus Staphylococcus aureus*, *P. aeruginosa Pseudomonas aeruginosa*, *C. albicans Candida albicans*, *A. fumigatus Aspergillus fumigatus*, *S. epidermidis Staphylococcus epidermidis*, *S. enterica Salmonella enterica*, *M. tuberculosis Mycobacterium tuberculosis*

## Conclusion and outlook

Long-term and disproportionate utilization of antibacterial and antifungal agents has resulted in the resistance of microorganisms to these agents as well as increase incidence of side effects on beneficial microbes. Accordingly, the development of nanoscale compounds with selective toxicity against bacterial strains or fungi is a viable approach due to the specific targeting of the selected microorganisms without the undiscriminating killing of the entire microbiota. Over the last decade, intensive efforts have been devoted to the fabrication of novel nanostructures or the conversion of conventional nanomaterials to those that possess a high affinity to specific microbes.

Selective toxicity may be implemented in most physiological systems by changing the surface functional groups and controlling their interactions with the cellular microenvironment. Given the unique cell wall characteristics of Gram-positive and Gram-negative bacteria, nanomaterials have been designed with surface-targeting moieties. These moieties include peptides, proteins, and antibodies that are capable of highly-specific interactions between the pathogens and the nanoagents. These agents kill the targeted bacteria via mechanisms such as cell membrane rupture and oxidative stress. Biomimetic strategies such as phage-guided antibacterial therapy and cell membrane coating for enhanced bacterial targeting have demonstrated enlightening results in several studies. Such strategies are rapidly becoming the avatar of contemporary pathogen targeting. Apart from bacteria, smart antifungal nanomaterials have been developed in which the targeting approaches were based on the recognition of the specific surface features of the destined fungus. The scientific community has devoted escalating efforts to the development of materials with selective pathogen-killing capability. This is achieved through rational design of the nanomaterials, and assessment of their selectivity using highly complex tissue/environments and/or animal models. These in-depth investigations pave the way for the emergence of infectious disease combating strategies with high specificity and reduced systematic toxicity. Especially given that most clinical infections are mainly treated empirically with antibiotics without prior identification of the associated pathogen. With advancements in real-time diagnosis of infectious pathogens, antibacterial nano-formulations with increased specificity are designed to reduce disease burdens with lower side effects. These experimental approaches, if translated into clinical applications, represent exciting alternatives to replace currently many over-used antibiotics. Nevertheless, these experimental approaches should be aligned with the principles of clinical trials and their associated regulations. One of the most important principles is the green biomaterials principle. In this regard, nanomaterials should possess bioactivity and demonstrate no adverse effects on the physiological system as well as the environment.

Synthetic and biosynthetic cationic polymers have been shown to possess microbial selectivity. The amino side chains in polypeptides (e.g., ε-polylysine) display more potent antimicrobial activity than those with guanidine side chains. Likewise, ethylenimines (e.g., polyethyleneimine) display better antibacterial activity than allylamines [129]. The cationic polymers (e.g., ε-polylysine and linear polyethyleneimine) exhibit bactericidal properties against antibiotic-resistant Gram-negative and Gram-positive bacteria and fungi by depolarizing the cytoplasmic membrane and disrupting biofilms [130]. In addition, electrostatic modification of bacterial surfaces using polyelectrolytes (e.g., polyethyleneimine and polyallylamine hydrochloride) is a convenient and versatile tool for biotechnological processes. Cationic polyelectrolytes such as polyethyleneimine or poly(allylamine) hydrochloride demonstrate specificity toward *Pseudomonas stutzeri*, a Gram-negative motile soil bacterium that causes opportunistic infections in immunocompromised patients [131]. It is important to consider the clinical regulations prior to proceeding with any type of experiment. These regulations vary in different countries. In addition, in vivo animal testing should only be performed after successful in vitro and ex vivo test outcomes.

Cationic micelles produced by cationic surfactants bearing amide moieties in spacers can efficiently kill Gram-negative microbes such as *E. coli* [131]. Increases in the degree of oligomerization can alter the antibacterial activity of these oligomeric surfactants. Electrically-conductive polymers (e.g., polyaniline and its derivatives, polypyrrole) doped with cationic surfactants such as hexameric quaternary ammonium surfactants, have been reported to possess selective antimicrobial activity against Gram-negative bacteria.

Activation of the host’s immune system is an important parameter to be considered for the practical application of antimicrobial nanomaterials against human infections and diseases. The anti-inflammatory properties of nanostructures can help balance inflammatory reactions triggered by bacteria cell death. This renders antimicrobial materials more attractive than antibiotics in the combat of multidrug-resistant infections. Pragmatically, however, the use of antimicrobial materials with specificity for the treatment of human infections still presents many challenges. Among them, understanding the immunological events associated with these nanosystems is vital for the rational design of biomedical materials with inherent anti-inflammatory and anti-infection properties, to simultaneously combat infections and modulate inflammation.

## Data Availability

Not applicable.

## Abbreviations

*A. baumannii*: *Acinetobacter baumannii*
AIE: Aggregation-induced emission
CARG: Cyclic 9-amino-acid peptide CARGGLKSC
CPP: Cell-penetrating peptide
*E. coli*: *Escherichia coli*
*E. faecalis*: *Enterococcus faecalis*
*F. tularensis*: *Francisella tularensis*
IL: Interleukin
INF-γ: Interferon-gamma
*K. pneumoniae*: *Klebsiella pneumoniae*
LPS: Lipopolysaccharide
*MRSA*: Methicillin-resistant *S. aureus*
NAG: *N*-Acetyl glucosamine
NAM: *N*-Acetyl muramic acid
OMV: Outer membrane vescicle
Onc112: A proline-rich antimicrobial peptide
PAMPs: Pathogen-associated molecular patterns
*P. aeruginosa*: *Pseudomonas aeruginosa*
ROS: Reactive oxygen species
*S. aureus*: *Staphylococcus aureus*
*S. agalactiae*: *Streptococcus agalactiae*
SNAPP: Structurally nanoengineered antimicrobial peptide polymer
TLR: Toll-like receptor
TNF-α: Tumor necrosis factor-α
*T. thermophilus*: *Thermus thermophilus*
