# Correction to: Bioengineered materials with selective antimicrobial toxicity in biomedicine

**DOI:** 10.1186/s40779-023-00466-8

**Published:** 2023-07-12

**Authors:** Pooyan Makvandi, Hao Song, Cynthia K. Y. Yiu, Rossella Sartorius, Ehsan Nazarzadeh Zare, Navid Rabiee, Wei-Xi Wu, Ana Cláudia Paiva-Santos, Xiang-Dong Wang, Cheng-Zhong Yu, Franklin R. Tay

**Affiliations:** 1 The Quzhou Affiliated Hospital of Wenzhou Medical University, Quzhou People’s Hospital, Quzhou, 324000 Zhejiang China; 2 Istituto Italiano di Tecnologia, Centre for Materials Interfaces, Pontedera, 56025 Italy; 3 Australian Institute for Bioengineering and Nanotechnology, The University of Queensland, Brisbane, QLD 4072 Australia; 4 Paediatric Dentistry and Orthodontics, Faculty of Dentistry, The University of Hong Kong, Prince Philip Dental Hospital, Hong Kong SAR, China; 5 Institute of Biochemistry and Cell Biology (IBBC), National Research Council (CNR), 80131 Naples, Italy; 6 School of Chemistry, Damghan University, Damghan, 36716-45667 Iran; 7 School of Engineering, Macquarie University, Sydney, NSW 2109 Australia; 8 Centre for Molecular Medicine and Innovative Therapeutics, Murdoch University, Perth, WA 6150 Australia; 9 Department of Pharmaceutical Technology, Faculty of Pharmacy of the University of Coimbra, University of Coimbra, 3000-548 Coimbra, Portugal; 10 REQUIMTE/LAQV, Group of Pharmaceutical Technology, Faculty of Pharmacy of the University of Coimbra, University of Coimbra, 3000-548 Coimbra, Portugal; 11 Department of Pulmonary and Critical Care Medicine, Zhongshan Hospital, Fudan University Shanghai Medical College, Shanghai, 200032 China; 12 School of Chemistry and Molecular Engineering, East China Normal University, Shanghai, 200241 China; 13 The Graduate School, Augusta University, Augusta, GA 30912 USA

**Correction to: Military Medical Research (2023) 10:8** 10.1186/s40779-023-00443-1

Following publication of the original article [1], it was noticed that the order of the affiliation 1 and affiliation 2 is incorrect. The correct order of the affiliations are shown below:

^1^ The Quzhou Affiliated Hospital of Wenzhou Medical University, Quzhou People’s Hospital, Quzhou 324000, Zhejiang, China

^2^ Istituto Italiano di Tecnologia, Centre for Materials Interfaces, Pontedera 56025, Italy

The original paper has been updated.

## References

[1] Makvandi P, Song H, Yiu CKY, Sartorius R, Zare EN, Rabiee N, et al. Bioengineered materials with selective antimicrobial toxicity in biomedicine. Mil Med Res. 2023;10(1):8. 10.1186/s40779-023-00443-1. 10.1186/s40779-023-00443-1 PMC9951506 36829246

